# Initial steps for the Portuguese Atlas of geographical variation in healthcare

**DOI:** 10.1007/s43999-023-00022-w

**Published:** 2023-04-07

**Authors:** Francisco von Hafe, Salomé Azevedo, José Fragata, Ana Rita Londral

**Affiliations:** 1 Value for Health CoLAB, NOVA Medical School, Lisbon, Portugal; 2 CHRC, Comprehensive Health Research Centre, NOVA Medical School, UNL, 1099-085 Lisbon, Portugal; 3 CEG-IST, Centre for Management Studies of Instituto Superior Técnico, Universidade de Lisboa, Lisbon, Portugal; 4 Department of Cardiothoracic Surgery, Hospital Santa Marta, Lisbon, Portugal Universidade Nova de Lisboa, Lisbon, Portugal

**Keywords:** Healthcare variations, Geographical variation, Practice variation, Regional health planning, Portuguese healthcare, Health policy

## Abstract

Non-clinical aspects contribute to differences in healthcare practices within each country, which may imply that some patients do not receive the care they need. In contrast, others may not benefit from the care that they receive. However, to the authors’ knowledge, only a few geographical variation studies with a national scope were recently conducted in Portugal. This study aimed to test if it was possible to conduct a geographical variation analysis in Portugal using publicly available data to compare the 18 districts and stimulate a debate around this topic. To achieve this goal, we first investigated the publicly available Portuguese National Health Service database (Transparency Portal) for data from activities and procedures that could be included in this analysis. Four were included: percentage of cesarian sections in total births, rate of hip surgeries within the first 48 h after admission in patients older than 65, rate of consumed antibiotics in the total drugs consumed, and percentage of elective surgeries. After retrieving the data, we mapped the results and computed the ratio of variation and the coefficient of variation. Finally, we discussed the results with medical doctors, public health researchers, and health economists. Results suggested geographical variation mainly in the rate of hip surgeries performed 48 h after admission (from 18.53% to 83.64%). Overall, the results highlighted the need for a national benchmarking system to span this analysis to other activities and initiate a broader discussion with patients, clinicians, providers, and policymakers.

## Introduction

Even living in the same country, different populations have different needs, cultures, values, and health risk behaviors. The variation in those characteristics may partially justify differences in access to healthcare services, healthcare processes, or outcomes [1]. Wennberg defined the variation that the mentioned factors cannot justify as *unwarranted variation*, i.e., “variation in the utilization of health care services that cannot be explained by variation in patient illness or patient preferences” [2]. Unwarranted variation may reflect, for example, differences in medical practices, healthcare organization, access to healthcare, or healthcare providers’ ability to generate demand [3]. The existence of geographical variation may imply that some patients are missing high-value interventions while others receive interventions that do not benefit or even harm them [3].

Geographical variation studies in Portugal are scarce [4]. In 2014, the Organisation for Economic Co-operation and Development (OECD) published a study on the geographic variation in healthcare that included Portugal [4]. Furthermore, the Portuguese National Institute of Health Doutor Ricardo Jorge and the Spanish Carlos III Health Institute published the Atlas of Cancer Mortality in Portugal and Spain 2003–2012 [5]. Moreover, several Ministry of Health bodies make available some platforms, such as the National Health Service (NHS) Transparency Portal [6] or the Central Administration of the Health System, I.P. Hospital Benchmarking [7], that provide information on healthcare practices in Portugal.

In February 2022, the Value for Health CoLAB (VoH CoLAB) initiated the development of the Portuguese Atlas of Variation in Healthcare. VoH CoLAB is a collaborative laboratory recognized by the Portuguese Foundation of Science and Technology (FCT), whose mission is to measure value in Health. Considering VoH CoLAB’s goal of contributing to increasing value in healthcare, understanding geographical variation in healthcare is crucial, as studying variation in healthcare highlights the opportunities to replace low with high-value activities [5, 6].

Oriented by Professor Sir J. A. Muir Gray, the geographical variation research for Portugal described in this brief communication aimed at answering the following question: “Can we start an unwarranted variation analysis with the publicly available healthcare system data?”. Our primary goals were to bring awareness to this topic and test whether publicly available data can be used to conduct an unwarranted variation study. Ultimately, to include Portugal in a growing list of countries publishing Atlases of Variation in Healthcare.

With the mentioned contributions, the VoH CoLAB team has developed the geographical variation analysis without additional funding.

## Methods

### Activities and procedures selection

We searched the publicly available Portuguese NHS Transparency Portal [6] for the data. This website, monthly updated by the Portuguese government [8], contains indicators of NHS access (such as the number of patients enrolled in primary healthcare or the number of hospital medical consultations), efficiency (for example, the average time before surgery or the number of workers by professional group), quality (including mortality from ischemic and hemorrhagic stroke or the percentage of hip fractures surgeries in the first 48 h after hospital admission), and Portuguese population health (such as diabetes incidence rate or the number of cancer screenings). Then, we conducted a literature search in the Atlas of Variation in Healthcare developed in other countries (Brazil [9], Spain [10], Australia [11], and the United Kingdom [12]) to select from the available activities and procedures on the NHS Transparency Portal the ones to include in our analysis. The selected activities and procedures should reflect important outcomes or high-impact processes [13]. Four different activities and procedures were selected: percentage of cesarian sections in total births, rate of hip surgeries within the first 48 h after admission in patients older than 65, rate of consumed antibiotics in the total drugs consumed, and percentage of elective surgeries.

### Data extraction

Data on healthcare activities and procedures was extracted from NHS Transparency Portal. The period of analysis was from January 2013 to December 2020. Data considers the location of the hospital or the health service where the care was provided.

### Data analysis

**Fig. 1:**
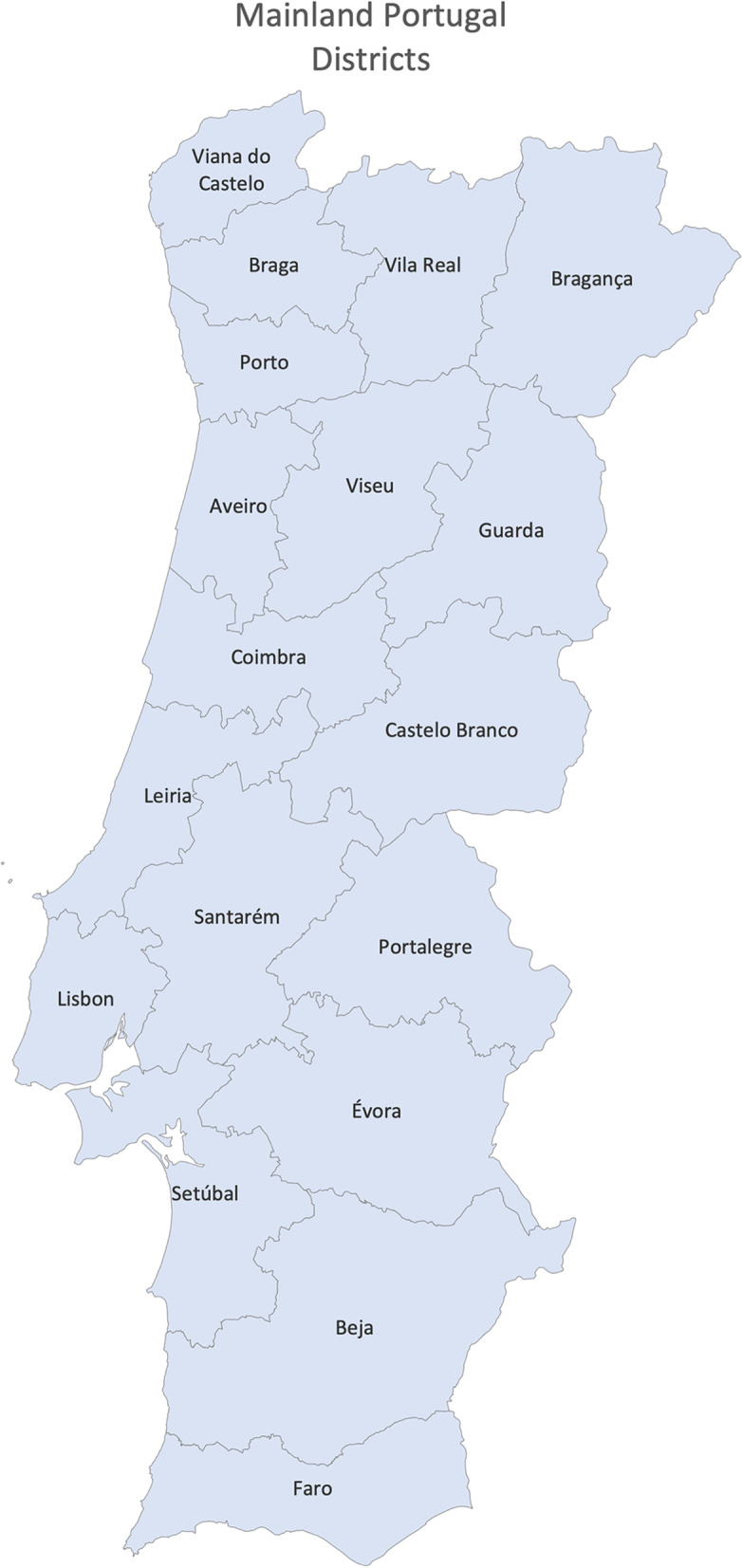
Mainland Portugal districts

After being extracted, data was aggregated at the district and Regional Health Administration (RHA) levels. Mainland Portugal is divided into 18 districts [14] (Fig. 1). RHAs are responsible for guaranteeing that the population in the area has access to healthcare, adapting the available resources to the population level, and fulfilling and enforcing health policies in their geographical area [15]. Mainland Portugal is divided into five RHA: North, Center, Lisboa and Vale do Tejo, Alentejo, and Algarve.

Considering the goal of this study to make large-scale comparisons of healthcare practices and activities across Portugal, we displayed data in line charts and maps, as we aimed to show the geographical variation and its magnitude [13]. Maps are color-classified using shades from light (lowest volume or rate) to dark blue (highest volume or rate) [9]. Moreover, we calculated two variation indicators: the ratio of variation, which determines how large the variation is by dividing the ninth decile by the first one [4], and the coefficient of variation, to determine the extent of variability to the population’s mean [16, 17].

Finally, doctors, public health researchers, and health economists initially discussed this brief communication’s results. Afterward, the results were discussed at the Wennberg International Collaborative & Swiss National Science Foundation Spring Synthesis Conference 2022 in Lucerne, Switzerland. The main goal was to discuss the obtained results with experts and understand the necessary steps to extend the analysis to other activities and conditions and how to disseminate the obtained results.

## Results

**Fig. 2:**
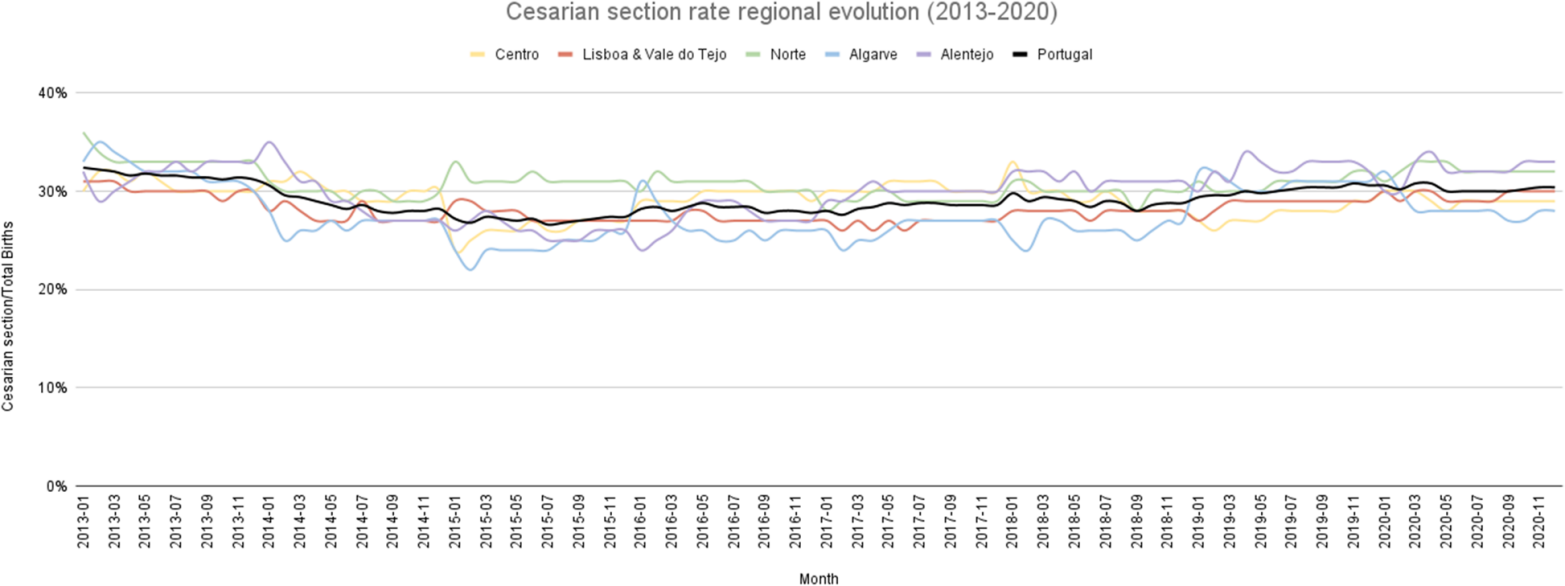
Percentage of cesarian sections in total births evolution per ​​Regional Health Administrations (2013—2020)

**Fig. 3:**
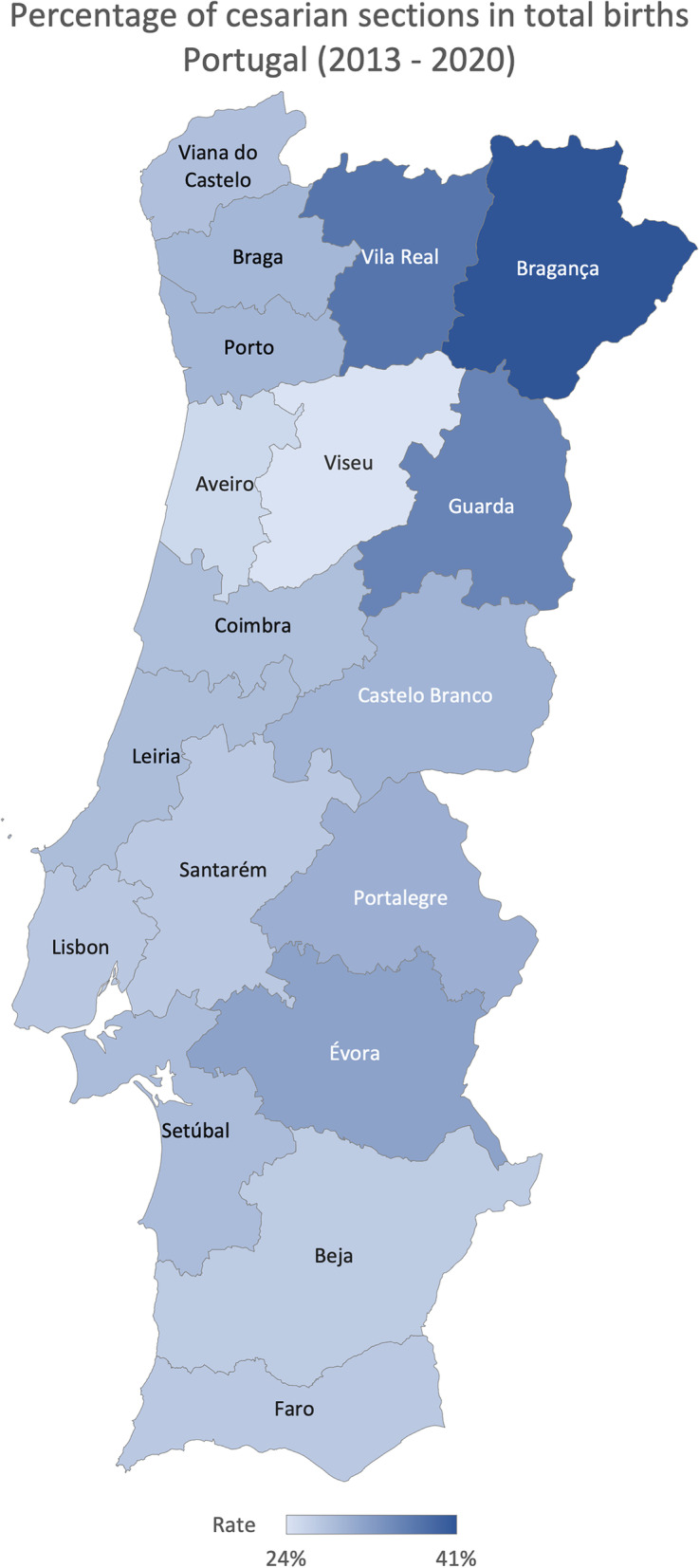
Percentage of cesarian sections in total births in Portugal (average 2013—2020)

**Fig. 4:**
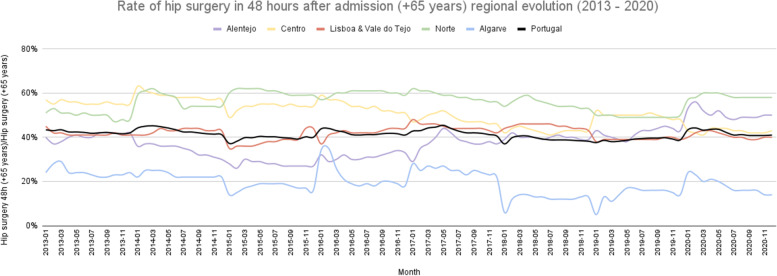
Rate of hip surgeries within the first 48 h after admission in patients older than 65 evolution per ​​Regional Health Administrations (2013—2020)

**Fig. 5:**
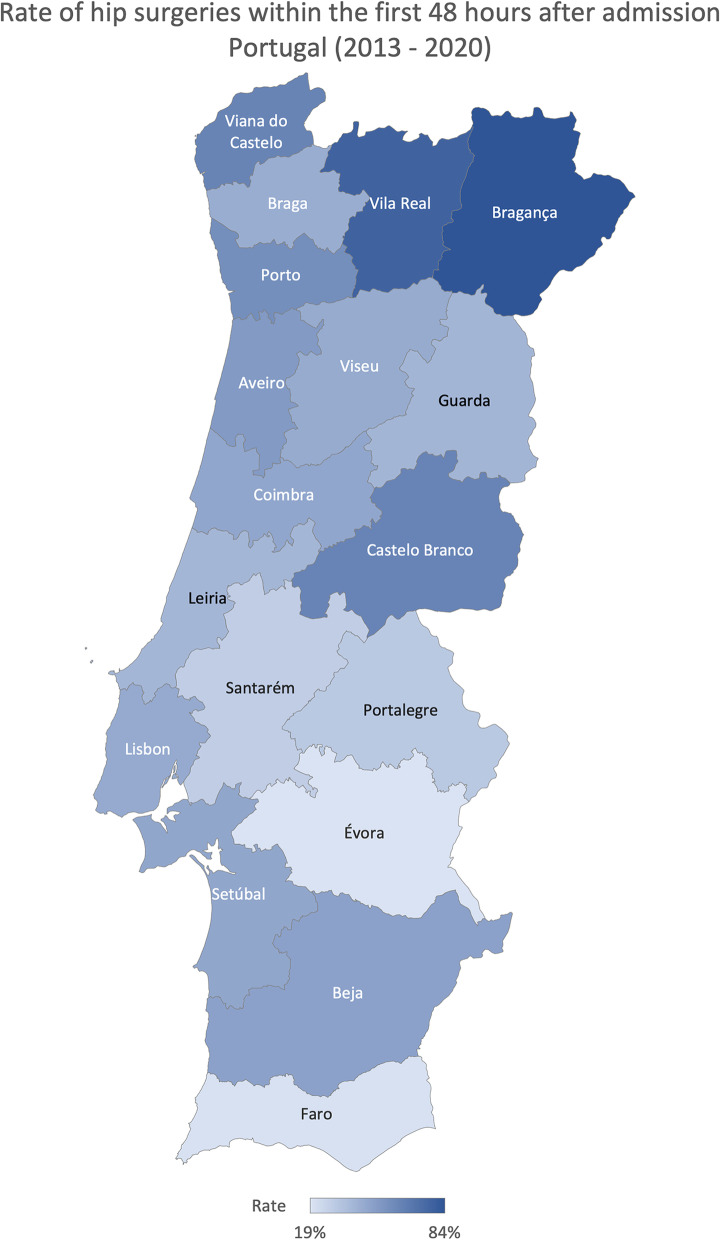
Rate of hip surgeries within the first 48 h after admission in Portugal (average 2013—2020)

In Portugal, the average cesarian section rate (total number of cesarian sections divided by the total number of births) was 29.42% between 2013 and 2020. The north region registered, on average, the highest rate (31.89%) and the Algarve the lowest one (27.66%) (Fig. 2). On average, between 2013 and 2020 district that registered lower cesarian section rate was Viseu (24.49%), and the highest was Bragança (41.31%) (Fig. 3). The ratio of variation was 1.50, and the coefficient of variation of 16.36% (Table 1). Focusing on the hip surgery 48 h after admission in patients older than 65 (number of hip surgeries conducted in the first 48 h after admission in patients over 65 divided by the number of hip surgeries conducted in patients over 65), North and Centre region reported a higher percentage of hip surgeries in the first 48 h (56.07% and 50.83%, respectively) on average (Fig. 4). All other RHA reported, on average, a rate lower than 50%, and Algarve registered the lowest (19.52%). Évora reported the lower percentage, 18.53%, and Bragança the highest, 83.64%, on average (Fig. 5). The ratio of variation is 4.00, and the coefficient of variation is 50.23% (Table 1).

**Table 1:** Summary of geographic variation

	Ratio of variation	Coefficient of Variation
Percentage of cesarian sections in total births	1.50	16.36%
Rate of hip surgeries within the first 48 h after admission in patients older than 65	4.00	50.23%
Rate of consumed antibiotics in the total drugs consumed	3.30	39.60%
Percentage of elective surgeries	1.26	8.26%

**Fig. 6:**
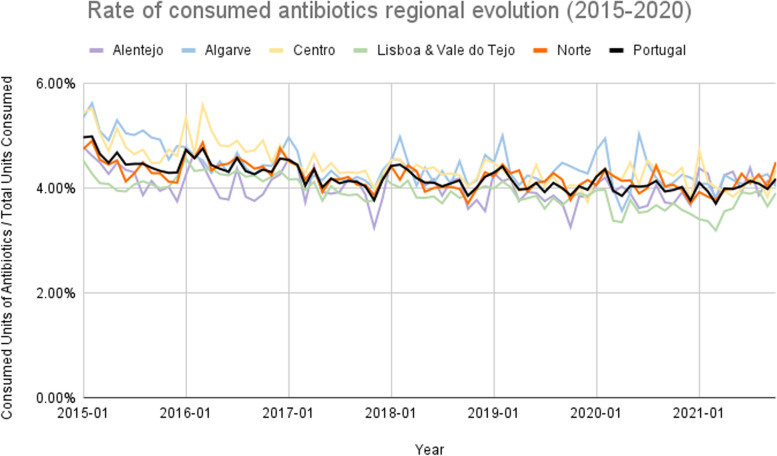
Rate of consumed antibiotics in the total drugs consumed evolution per ​​Regional Health Administrations (2015—2020)

**Fig. 7:**
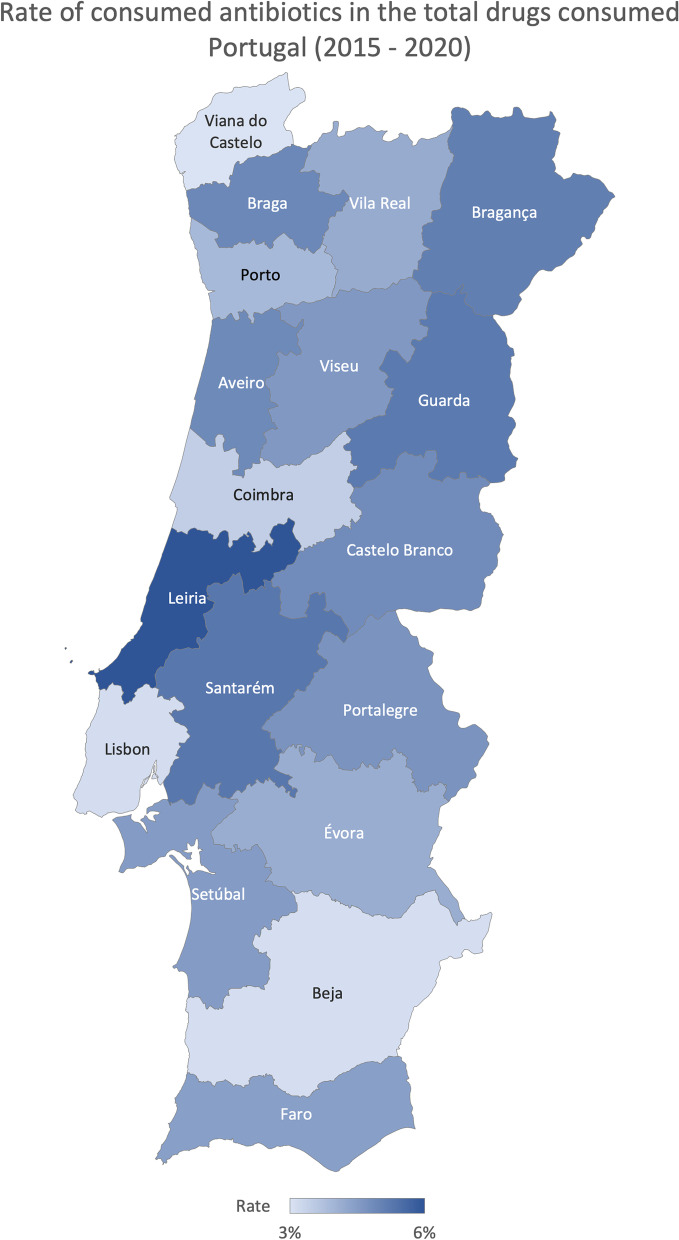
Rate of consumed antibiotics in the total drugs consumed in Portugal (average 2015—2020)

**Fig. 8:**
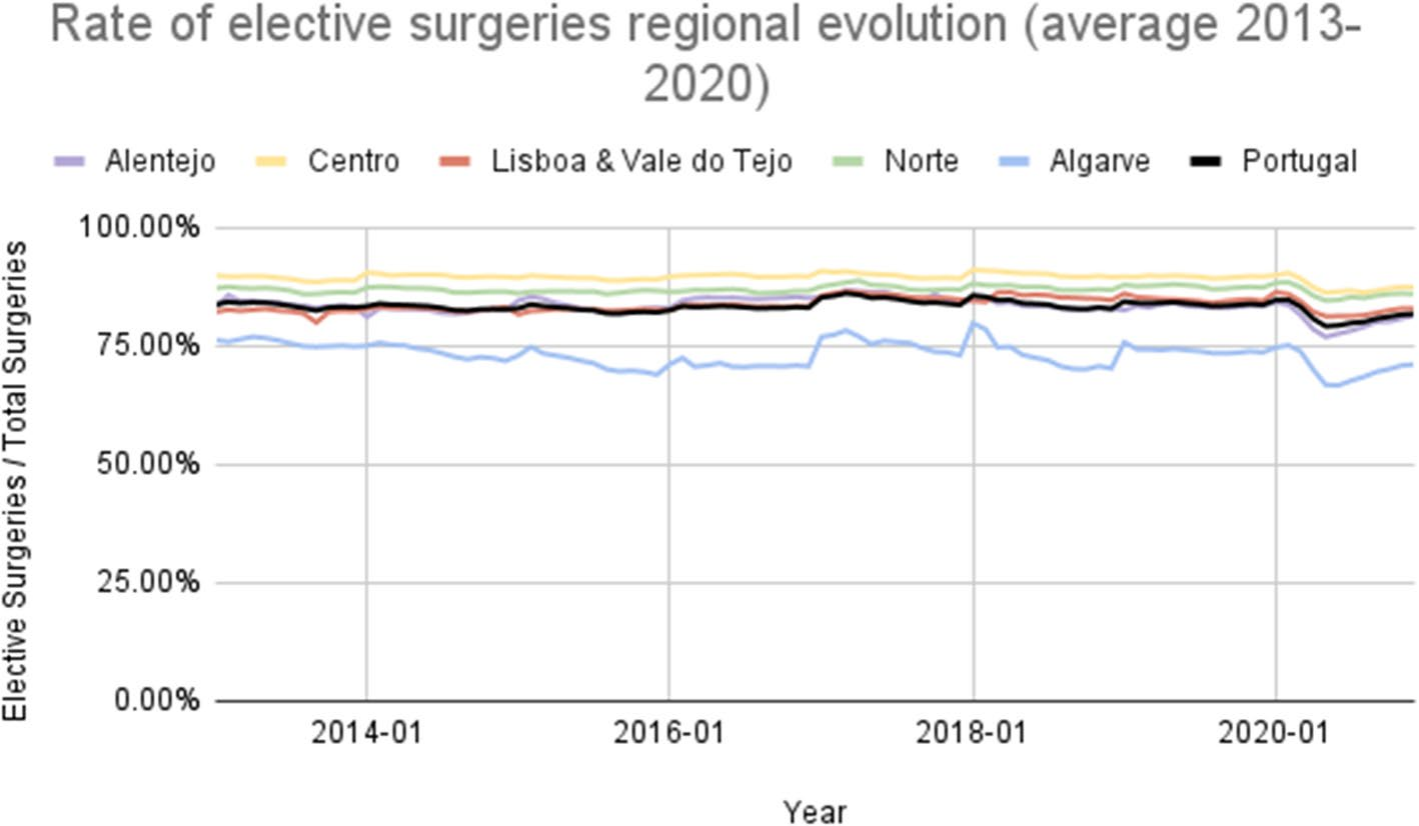
Percentage of elective surgeries evolution per ​​Regional Health Administrations (2013—2020)

**Fig. 9:**
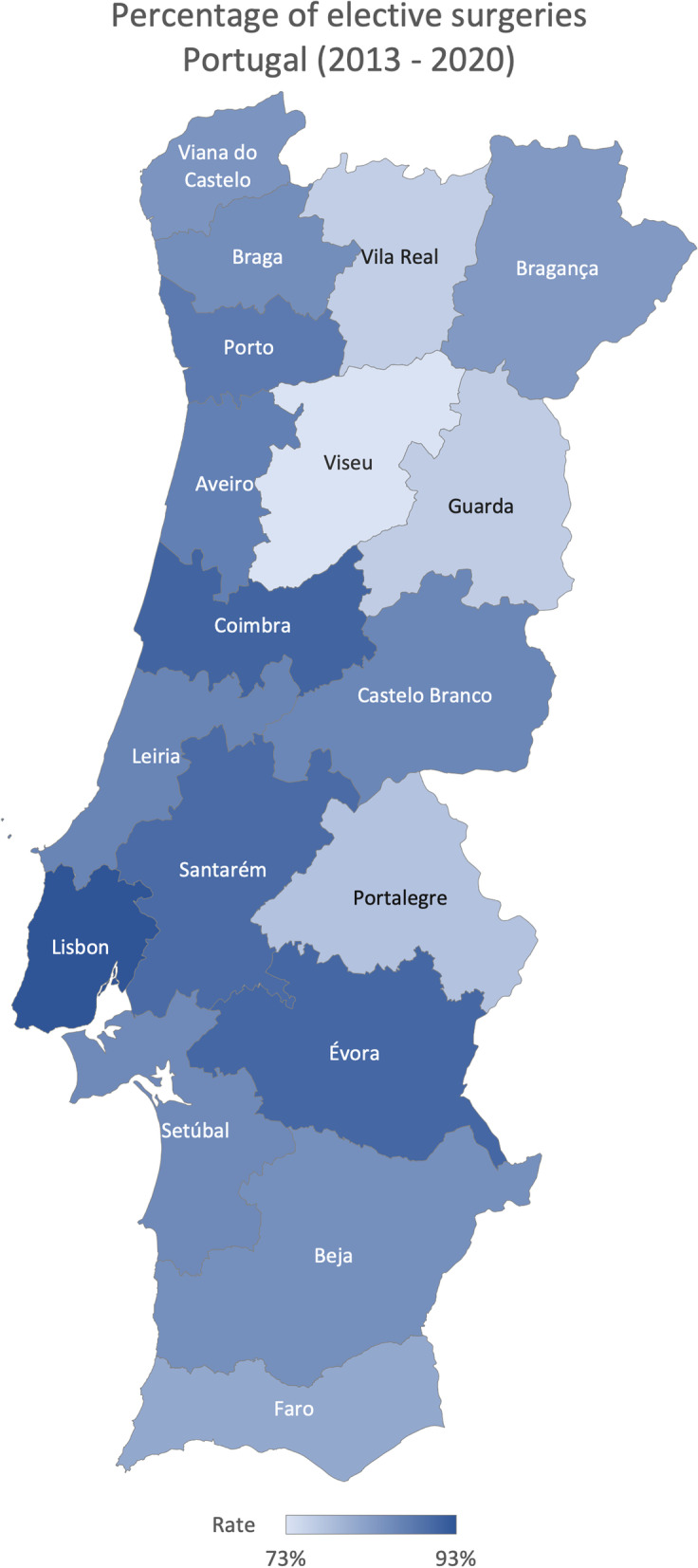
Percentage of elective surgeries in Portugal (average 2013—2020)

The average percentage of antibiotics consumed (consumed units of antibiotics divided by the total units consumed) in the total consumed drugs was 4.18% in Portugal from 2015 to 2020. The Algarve region registered the highest percentage between 2015 and 2020 (4.45%), and Lisboa and Vale do Tejo had the lowest, 3.98%, on average (Fig. 6). On average, Leira registered the higher percentage, 6.15%, and Viana do Castelo the lowest, 2.88% (Fig. 7), with a ratio of variation of 3.30 and a coefficient of variation of 39.60% (Table 1). Finally, the percentage of elective surgeries (number of elective surgeries divided by the total number of surgeries) in Portugal from 2013 to 2020 was, on average, 85.86%. Centre region reported the highest rate (89.45%) and the Algarve’s lowest rate (73.11%), on average (Fig. 8). The Coimbra district registered the highest percentage, 92.80%, and the Faro the lowest, 73.11%, on average (Fig. 9), with a ratio of 1.26 and a coefficient of variation of 8.26% (Table 1).

## Discussion

This brief communication aimed to bring awareness to the geographic variation in healthcare topic and to assess whether it was possible to study it with publicly available data. We observed higher variation in hip surgeries within the first 48 h after admission in patients older than 65, followed by the rate of antibiotics consumed in the total drugs consumed, and the percentage of cesarian sections in total births. These results should be seen as a first step to initiate a study to identify unwarranted variation in clinical practice in Portugal.

Overall, comparing districts instead of RHA allowed us to understand variations that would only be noticed if we compared RHA. For most activities and procedures, district variation was higher than RHA variation. This highlights the importance of assessing variation at the district level [9].

This study has limitations that should be considered when analyzing the results. First, only public hospitals were included in the analysis, as only public hospitals’ information was available on the NHS Transparency Portal. Second, data was not adjusted for population characteristics as hospitals provide care to patients living outside the hospital’s district, so it is impossible to determine the patients’ district of residence with the available data. Adjusting for the population’s characteristics, such as sex and age, partially rules out that the found variation is not entirely caused by different population characteristics, allowing for comparison between districts with different age and sex structures. Finally, we considered the hospital location rather than the patient one due to data availability. However, using the patient’s place of residence could allow us to study if there is inequity in the access to healthcare services. Overall, these limitations limit the comparison of these study results with other geographical variation studies conducted in Portugal, as other studies standardize the presented rates or use the patient’s place of residence rather than the health facility [4, 18]. However, one study about the variation of cesarian sections (between 2008 and 2012 in Portuguese public hospitals) that considered the healthcare facility’s location also found that most cesarian sections occur in the North of Portugal [19].

After discussing the results with doctors and public health researchers, they agreed on the need for future studies to understand the reasons behind the observed variation. After conducting those studies, they believe it will be possible to create a benchmarking system in Portugal that helps to set “gold standards” for clinical practice, as observed for specific conditions in the United Kingdom or Australia [1, 20]. When the Portuguese Health Atlas of Variation is concluded, we expect to disseminate the results to the Portuguese population, mainly focusing on healthcare professionals and decision-makers. Hopefully, this study’s results will trigger the development of other studies that, for example, use patient-level data to produce guidelines that consider patients’ needs and characteristics in the variation analysis of the district or RHA-level healthcare practice. When developing those guidelines, contributions from patients, clinicians, healthcare providers, and policymakers should be considered. Although countries can learn from other countries practices, research on each country’s population and healthcare system is necessary [9].

In conclusion, it was not possible to initiate an unwarranted variation study with publicly available data for the Portuguese context due to the limitations mentioned earlier. However, we believe these initial results will motivate others to pursue studies related to geographical variation. We are currently working on the Portuguese Atlas of Variation in the National Health Service. In this Atlas, we are using patient-level data from public hospitals in Portugal, which allows to control data for sex and age and use patients’ place of residence. The results of our current work will promote a more informed discussion on the extent to which variation is unwarranted, its causes, and strategies to address it.
